# Bioinspired Polymers: Transformative Applications in Biomedicine and Regenerative Medicine

**DOI:** 10.3390/life13081673

**Published:** 2023-08-01

**Authors:** Hossein Omidian, Renae L. Wilson, Niloofar Babanejad

**Affiliations:** Barry and Judy Silverman College of Pharmacy, Nova Southeastern University, Fort Lauderdale, FL 33328, USA; rw1273@mynsu.nova.edu (R.L.W.); nb1141@mynsu.nova.edu (N.B.)

**Keywords:** bioinspired polymers, biomedical engineering, tissue engineering, regenerative medicine, biomedicine

## Abstract

Bioinspired polymers have emerged as a promising field in biomaterials research, offering innovative solutions for various applications in biomedical engineering. This manuscript provides an overview of the advancements and potential of bioinspired polymers in tissue engineering, regenerative medicine, and biomedicine. The manuscript discusses their role in enhancing mechanical properties, mimicking the extracellular matrix, incorporating hydrophobic particles for self-healing abilities, and improving stability. Additionally, it explores their applications in antibacterial properties, optical and sensing applications, cancer therapy, and wound healing. The manuscript emphasizes the significance of bioinspired polymers in expanding biomedical applications, addressing healthcare challenges, and improving outcomes. By highlighting these achievements, this manuscript highlights the transformative impact of bioinspired polymers in biomedical engineering and sets the stage for further research and development in the field.

## 1. Introduction

The awe-inspiring properties exhibited by various bio species, such as geckos, mussels, and tree frogs, have ignited the curiosity of scientists and inspired the development of novel biomimetic polymers and materials with remarkable adhesion capabilities. These bio-inspired adhesives hold tremendous potential for a wide array of applications across diverse industries, including medical, robotics, and construction.

Geckos, with their incredible ability to cling to surfaces, owe their adhesive prowess to the hierarchical structure of their feet, involving micro/nano fibrillar structures and van der Waals forces. Scientists have successfully replicated this structure to create synthetic dry adhesives that demonstrate outstanding adhesion strength, directional adhesion, and structural robustness. Such materials find invaluable applications in precision industries like semiconductor manufacturing and display production, enabling precise manipulation of large-area substrates without the need for external force application [1,2].

Drawing inspiration from mussels, which achieve robust attachment underwater through mussel foot proteins (mfps) decorated with 3,4-dihydroxyphenyl-l-alanine (DOPA), researchers have developed mussel-inspired functional materials. Incorporating catechol groups into bio-inspired polymers, such as polyethyleneimine-catechol and chitosan-catechol, has yielded wet-resistant adhesives with excellent hemostatic abilities and tissue adhesion [3].

In the pursuit of wet adhesion solutions, tree frogs and certain insects have inspired the creation of eco-friendly adhesives that capitalize on capillary forces and surface design. An example is a green soybean meal-based adhesive integrated with modified kenaf fibers, showcasing robust mechanical interlocking and multiple chemical crosslinking abilities. This adhesive offers a promising alternative to formaldehyde-based adhesives in the wood industry [4].

Hydrophilic drug-in-adhesive patches using PHI-cat and HA have been developed for transdermal drug delivery, combining effective drug release with excellent adhesion properties [5]. Mussel-inspired dopamine chemistry has led to antimicrobial zwitterionic polymer coatings, reducing bacterial adhesion, and improving the performance of implantable biomedical devices [6]. Polydopamine coatings and catechol conjugation show promise in regenerative medicine, especially in drug delivery systems and tissue regeneration [7]. Silver-releasing antibacterial hydrogels and tannin-inspired bioadhesives derived from tannic acid and gelatin offer antibacterial properties suitable for wound closure, tissue sealant, and drug delivery [8,9]. Synthetic adhesives mimicking gecko setae and mussel holdfasts offer enhanced adhesion properties, extending applications beyond traditional pressure-sensitive adhesives [10]. Native and engineered silk biomaterials with improved drug loading and release properties hold great potential for drug delivery systems [11].

By harnessing the adhesive properties of mussels, zwitterionic polymer coatings have been developed to enhance the performance and longevity of implantable biomedical devices [6]. Biohybrid nanoreactors responsive to glucose enable targeted antimicrobial activity for treating bacterial infections [12]. Crosslinked nanoparticles, influenced by bioinspired approaches, demonstrate improved stability and controlled drug release properties for targeted and sustained drug delivery [13]. Mussel-inspired chemistry has also found application in glycosylated and PEGylated carbon nanotube composites, stabilization of alginate hydrogel fibers, modification of natural polysaccharides, and development of tannic acid-based polymer brushes to inhibit bacterial adhesion [14,15,16,17]. 

Studies highlight functionalized polymers to improve mechanical properties and durability in cell culture applications, such as anatomically and biologically inspired PEG-based hydrogels, mussel-inspired catechol chemistry, and composite hydrogels with hydrophobic particles [18]. Anatomically and biologically inspired PEG-based hydrogels for heart valve tissue engineering mimic the extracellular matrix, enhancing mechanical performance and promoting cell behavior [19]. Mussel-inspired catechol chemistry improves the mechanical properties of alginate hydrogel fibers, seeking enhanced stability and tensile strength for tissue scaffolds [15]. Additionally, the synthesis of composite hydrogels incorporating hydrophobic particles provides insights into self-healing ability, mechanical properties, and stability mechanisms, facilitating the design of robust materials [20]. 

Inspired by natural antimicrobial peptides, synthetic antimicrobial polymers have been developed with broad-spectrum antibacterial activity and reduced bacterial resistance [21]. These polymers mimic the structure and function of natural peptides, offering a new approach to combat antibiotic-resistant bacteria. Additionally, bioinspired surface modifications, such as nanostructured surfaces and nanopatterned coatings, have been explored to inhibit bacterial attachment and biofilm formation, providing effective strategies for preventing infections [22].

Biomimetic photonic materials, inspired by structural colors found in nature, have been developed for various applications, including colorimetric sensing, chemical detection, and optical coatings [23]. These materials replicate intricate nanostructures found in butterfly wings or peacock feathers, resulting in vibrant and tunable colors without the use of dyes or pigments. Furthermore, bioinspired sensors based on biological receptors, such as antibodies and enzymes, integrated with polymer matrices, offer sensitive and selective detection of target analytes for medical diagnostics, environmental monitoring, and food safety [24].

Nanoparticles functionalized with targeting ligands, such as antibodies or peptides, can specifically recognize cancer cells and deliver therapeutic agents directly to the tumor site, minimizing side effects [25]. Stimuli-responsive polymers have also been developed to achieve controlled drug release in response to specific triggers, such as pH, temperature, or enzymatic activity [26]. These systems provide spatiotemporal control over drug release, optimizing therapeutic efficacy and minimizing toxicity.

Scaffold materials mimicking the extracellular matrix have been developed to provide structural support and promote cell adhesion, proliferation, and differentiation [27]. These biomimetic scaffolds offer a conducive microenvironment for tissue regeneration, enabling the repair of damaged or diseased tissues. Additionally, bioinspired self-healing hydrogels, such as self-healing hydrogels and injectable hydrogels, have been explored for wound healing applications, providing mechanical support, promoting cell migration, and facilitating tissue regeneration [28].

This manuscript aims to provide an overview of the transformative potential of bioinspired polymers in various fields, including tissue engineering, regenerative medicine, and biomedicine. It will cover different aspects of bioinspired polymers, such as their role in enhancing mechanical properties and durability in cell culture applications, mimicking the extracellular matrix for tissue engineering, improving stability and tensile strength for tissue scaffolds, and incorporating hydrophobic particles for self-healing ability.

## 2. Polymer Synthesis

Bioinspired polymers have emerged as a captivating area of research, offering promising opportunities for developing advanced materials with improved properties and functionalities. Researchers have employed various reaction synthesis, polymerization, and crosslinking techniques to create bioinspired polymers that emulate the structures and properties found in biological systems, drawing inspiration from nature. These innovative approaches have demonstrated significant potential in diverse biomedical applications, such as drug delivery, tissue engineering, and antimicrobial coatings.

**Figure 1:**
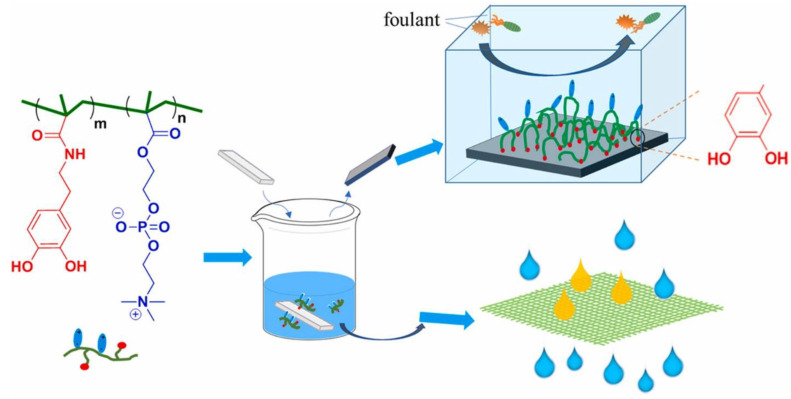
Dopamine chemistry in the field of surface chemistry as an adhesion base for the synthesis of antifouling coatings [29].

One noteworthy technique involves synthesizing bioinspired polymers through dopamine chemistry. Dopamine-mediated copolymers have been developed to combine adhesion and antifouling properties, making them well-suited for surface modification applications (Figure 1) [29]. These copolymers not only display enhanced antifouling capabilities but also offer oil-water separation capabilities. The incorporation of dopamine chemistry into the polymer synthesis process opens up new possibilities for creating functional surfaces with improved performance.

**Figure 2:**
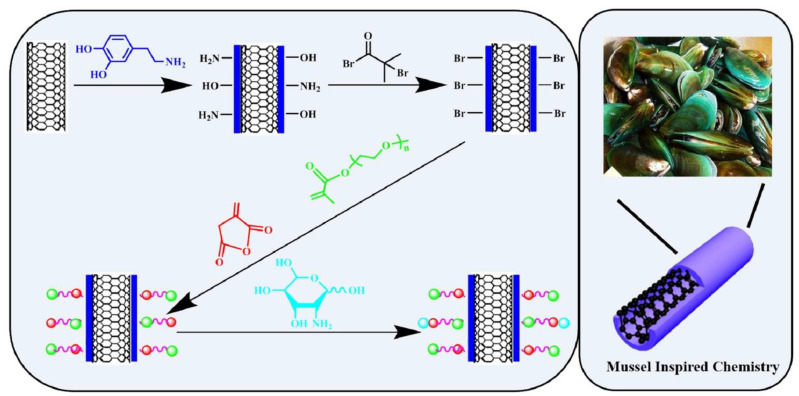
Glycosylation and PEGylation techniques on carbon nanotubes for potential biomedical applications [14].

Another approach involves the use of mussel-inspired chemistry for polymer modification. Mussel-inspired strategies have been employed to modify natural polysaccharides, such as alginate, in biomedical applications, expanding their uses in tissue engineering, wound healing, drug delivery, and biosensors [16]. By employing glycosylation and PEGylation techniques, researchers have successfully enhanced the dispersibility and cytocompatibility of carbon nanotubes for potential biomedical applications (Figure 2) [14]. Additionally, mussel-inspired surface molecular imprinting polymers on reduced graphene oxide have demonstrated excellent electrocatalytic activity and selectivity, enabling sensitive and selective electrochemical sensing [30].

**Figure 3:**
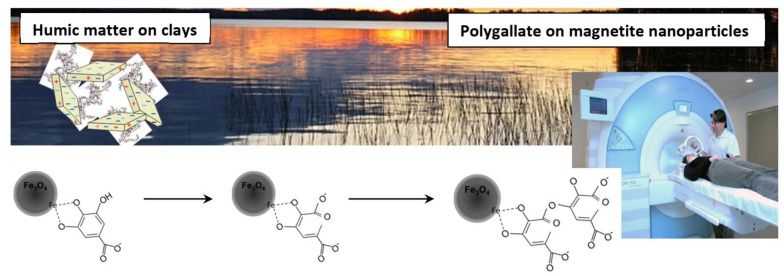
Synthesis of carboxylated core-shell magnetite nanoparticles with a polygallate (PGA) coating [32].

Polymerization mechanisms also play a crucial role in the synthesis of bioinspired polymers. Tannic acid-based star polymers synthesized via electrochemically mediated atom transfer radical polymerization (ATRP) offer controlled polymerization and well-defined structures for various biomedical applications, including drug delivery, antifouling coatings, and antimicrobial applications [31]. Furthermore, carboxylated core-shell magnetite nanoparticles with a polygallate (PGA) coating have been synthesized using polymerization mechanisms, contributing to their antioxidant capacity and potential applications in MRI contrasting, magnetic hyperthermia treatments, and drug delivery (Figure 3) [32].

**Figure 4:**
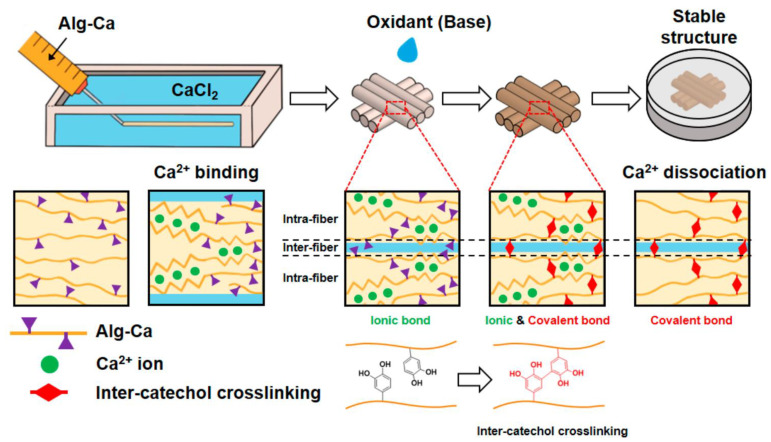
Schematic illustration of preparation of wet-spun Alg-Ca fibers and their 3D fibrous constructs with oxidative treatment. The fibers and their 3D structure are fabricated using initial ionic bonds followed by inter-catechol crosslinking even after dissociation of ionic bond [15].

The crosslinking of bioinspired polymers is another crucial aspect that impacts their performance and functionality. Alginate hydrogel fibers stabilized using mussel-inspired catechol chemistry have demonstrated remarkable mechanical strength, making them suitable for designing tissue scaffolds (Figure 4) [15]. The crosslinked structure of these hydrogel fibers ensures shape fidelity and provides an optimal environment for tissue regeneration.

## 3. Adhesion

A diverse range of bioinspired materials, including hydrophilic drug-in-adhesive patches, dual functional coatings, mussel-inspired biomaterials, tannin-inspired gelatin bioadhesives, synthetic polymer-based dry adhesives, and biodegradable surgical glues have demonstrated exceptional adhesion properties. Additionally, the development of composite membranes, gecko-inspired tissue adhesives, polydopamine coatings, and supramolecular hydrogels showcase the versatility and potential of bioinspired materials in addressing specific challenges and improving performance in various applications.

One area of focus is the development of hydrophilic drug-in-adhesive patches composed of PHI-cat (polyhexylisocyanate-catechol) and HA (hyaluronic acid) for transdermal drug delivery systems. These patches not only demonstrate excellent adhesion to human skin but also exhibit good stability due to their PHI-cat and HA content. [5]. Similarly, a dual functional coating based on dopamine chemistry has been designed to create a zwitterionic polymer coating with antifouling and antimicrobial properties for implantable biomedical devices. This coating effectively reduces bacterial adhesion and allows for the in-situ deposition of antimicrobial silver nanoparticles [6]. Copolymerization of the catechol- containing dopamine methacrylamide (DMA) and bioinspired zwitterionic 2-methacryloyloxyethyl phosphorylcholine (MPC) provided stability on an antifouling surface that was covalently grafted onto an amino (-NH(2)) rich polyethyleneimine (PEI)/polydopamine (PDA) codeposit surface [6].

Mussel-inspired biomaterials, such as polydopamine coating and catechol conjugation, hold great promise in regenerative medicine, tissue engineering, and hemostatic control. This is due to the P(DMA-co-MPC) copolymer-modified substrates [7]. Furthermore, antibacterial hydrogels synthesized with catechol-containing PEG polymers exhibit sustained silver release that inhibits bacterial growth, and resist cell attachment, making them suitable for antibacterial biomaterial coatings and tissue adhesives [8].

Liquid-free ionoelastomers inspired by mussels have emerged as remarkable materials with universal adhesion, underwater self-healing capabilities, sensing ability, and flame retardancy. These materials find applications in coatings, adhesives, biomedical engineering, wearable electronics, and human-machine interfaces [33]. Additionally, recombinant silk biomaterials, including recombinant silks, offer tailored drug delivery systems with advantages such as processability, self-assembly, drug loading capacity, and customizable physicochemical properties [11].

Tannin-inspired gelatin bioadhesives derived from tannic acid and gelatin exhibit wet tissue adhesion, antimicrobial properties, controllable degradation, and cytocompatibility, making them suitable for wound closure, tissue sealants, hemostasis, antimicrobial treatments, and drug delivery [9]. Similarly, 2D polydopamine nanosheets serve as functional surfaces for grafting polymer brushes, showcasing lateral integrity, robustness, and cell-adhesion-promoting properties. These functional surfaces find applications in biomaterial coatings and nonfouling behavior [34].

Muco-adhesive polymers play a significant role in controlled drug delivery systems, and recent advancements have focused on enhancing their performance and efficacy in various administration routes. Bio-inspired muco-adhesive polymers offer improved drug delivery performance [35]. Citrate-based (POEC-d) tissue adhesives surpass commercial fibrin glue in wet tissue adhesion during surgeries due to their water solubility, biodegradability, and good adhesive properties [36].

Synthetic polymer-based dry adhesives that mimic gecko adhesive properties have been developed and can be tailored to specific industry needs. These adhesives exhibit anisotropic adhesion through a scalable fabrication process and provide insights from the nanoscale tape-peeling model [33]. Loop-conformation triblock copolymers consisting of two end blocks of catechol-anchoring groups and a loop of poly(ethylene oxide) (PEO) midblock provide robust lubricating and antifouling properties with low friction coefficients and reduced cell adhesion, thereby finding potential applications in specialized biomedical coatings and surface engineering [37].

Biodegradable surgical glue, known as TAPE, which is a combination of Tannic Acid (TA) and biopolymer, Poly(Ethylene) glycol (PEG), demonstrates enhanced hemostatic ability and water-resistant adhesion strength compared to commercial tissue adhesives. This innovation addresses challenges in wet environments and finds applications in hemostatic glues and drug delivery systems [38]. Furthermore, a hybrid adhesive inspired by gecko and mussel adhesion combines nanofabricated polymer pillars with synthetic mussel adhesive proteins, resulting in increased wet adhesion and reversibility on various surfaces and environments nearly 15-fold [10].

A composite polymer membrane for joint wound dressings combined alginate-based helical fibers and polyacrylamide/gelatin (PAM-Gel), offering better mechanical properties such as adhesion strength, stretchability, biocompatibility, high transparency along with controlled drug release. Inspired by the endometrium’s spiral arteries, this membrane provides functional solutions for joint wound dressings [39]. Similarly, gecko-inspired tissue adhesive utilizes nanotopography and biocompatible surface coating to enhance tissue adhesion, offering potential applications in wound sealing and drug delivery as an alternative to sutures or staples [40].

Mussel-inspired polydopamine coating coupled with a PHA (polyhydroxyalkanoate) delivery system shows promise in improving oral mucosal treatment. This research investigates functionalization, adhesion properties, and biocompatibility for treating oral conditions [41]. A material-independent surface coating enables the feeder-free expansion of human pluripotent stem cells (hPSCs) for tissue engineering applications. The polydopamine-mediated immobilization of VN peptides facilitates adhesion, proliferation, and colony formation of hPSCs [42].

Tannic acid (TA) primers have enabled the graft-polymerization of functional polymer brushes with bactericidal properties and resistance against bacterial adhesion. These coatings offer reduced microfouling and macrofouling, providing anti-fouling solutions [17]. Similarly, bio-inspired polymer coatings of methoxy-terminated poly(ethylene glycol) (mPEG) conjugated to the adhesive amino acid l-3,4-dihydroxyphenylalanine (DOPA) on titanium surfaces exhibit superior resistance to protein and cell fouling, offering promising solutions for marine antifouling coatings [43]. Additionally, polydopamine (PDA) coatings on implant surfaces enhance cell adhesion, proliferation, and osteogenic differentiation, leading to improved osseointegration performance [44].

Polymer microparticles with controllable surface roughness demonstrate strong adhesion to the intestinal mucosa, enabling enhanced drug loading and controlled release kinetics for drug delivery applications [45]. Supramolecular hydrogels inspired by pyrogallol-bearing proteins found in ascidians exhibit fast gelation ability and hold promise for biomedical applications, including topical drug delivery and wearable electronic devices [46]. Furthermore, bioinspired hydrogels incorporating catechol-containing compounds enhance mucoadhesion in wet conditions, offering potential applications in drug delivery and tissue engineering [47].

Biodegradable microfibrous scaffolds with thermoswitchable properties regulate endothelial cell adhesion, potentially enabling targeted cell recruitment in regenerative medicine applications [48]. Mussel-inspired underwater adhesive polymers demonstrate vast potential in commercial applications such as intermediates, anti-biofouling, self-healing hydrogels, biological adhesives, and drug delivery systems [49]. Lastly, biohybrid composite materials combining low-fouling polymer hydrogel networks with encapsulated antimicrobial enzymes exhibit passive low-fouling properties and active antimicrobial activity, effectively reducing bacterial adherence in industrial applications [50].

## 4. Mechanical Properties

The quest to develop materials with enhanced mechanical properties with biomaterials is paving the way for applications in various fields. The following entries highlight the mechanical property aspects of these bioinspired materials, shedding light on their potential and significance.

One notable example is the composite hydrogel of Fmoc-RGD hydrogelator and chitosan, which demonstrates enhanced mechanical properties, durability, and thixotropic behavior, making it an ideal scaffold for 2D and 3D cell cultures [18]. The incorporation of these bioinspired components enhances the mechanical integrity of the hydrogel, providing structural support for cell growth and tissue engineering applications.

Supramolecular polymer hydrogels inspired by sea cucumbers doped with Ca(2+) exhibit remarkable stimuli-responsive mechanical properties, high strength, stretchability, toughness, and self-healing ability [51]. These bioinspired hydrogels offer exciting possibilities for applications requiring materials with superior mechanical performance, such as drug delivery systems and tissue engineering scaffolds.

Kneading-dough-inspired hydrogels disperse hydrophobic particles, resulting in self-healing hydrogels with tunable mechanical properties [20]. This unique characteristic makes them highly valuable for applications in drug delivery and biosensor development, where mechanical robustness and self-repair capabilities are essential.

Stabilization of alginate hydrogel fibers using catechol chemistry enhances their mechanical properties, enabling their application in fibrous-type tissue scaffolds and bioink materials [15]. These bioinspired fibers provide mechanical strength and structural integrity, crucial for tissue engineering and 3D bioprinting applications.

Anisotropic poly(ethylene glycol) hydrogels with biomimetic properties have been developed for heart valve tissue engineering, mimicking the anisotropic behavior of native valve leaflets [19]. The incorporation of cell-adhesive peptides and collagenase-degradable peptides influences valvular interstitial cell behavior, ensuring appropriate mechanical responses and functionality of the tissue-engineered heart valves.

## 5. Antibacterial and Antifouling Properties

Bioinspired polymers have emerged as a versatile class of materials with multifunctional properties, including remarkable antibacterial activity. These polymers draw inspiration from natural processes and organisms to design surfaces, coatings, and materials that exhibit potent antibacterial properties. In this section, we will focus on the antibacterial aspects of bioinspired polymers, highlighting recent advancements and their potential applications in various fields.

Poly(oxonorbornene)-based synthetic mimics of antimicrobial peptides (SMAMPs) have demonstrated antimicrobial activity against bacterial strains with low toxicity to human cells, making them potential candidates for clinical applications [52]. These polymers offer a promising approach to combat bacterial infections while minimizing harmful effects on human cells.

Glucose-responsive biohybrid glucose oxidase-loaded semipermeable polymersome antimicrobial nanoreactors have shown significant antibacterial properties against bacterial pathogens, including methicillin-resistant Staphylococcus aureus (MRSA) [12]. These nanoreactors hold potential as antimicrobial biomaterials for the treatment of bacterial infections, offering a targeted and effective therapeutic strategy.

Chitosan-functionalized RGD-based hydrogels have exhibited improved mechanical properties, durability, and antibacterial activity [18]. The composite hydrogel holds promise as a scaffold for 2D and 3D cell cultures in tissue engineering and cell culture applications, providing a favorable environment for cell growth while combating bacterial contamination.

Metal-biomolecule complexes based on phenylalanine have been developed to detect and remove hazardous substances from water, showing antimicrobial properties that suggest potential use in wastewater treatment [21]. These complexes, such as the Cu(ii)-aspartame coordination polymer, offer a dual functionality of pollutant removal and antimicrobial action.

Polymeric conjugates of polymyxin B have exhibited reduced cytotoxicity and preserved antimicrobial properties against Pseudomonas aeruginosa [22]. These conjugates hold promise as carriers for controlled drug delivery, offering improved antibiotic efficacy and reduced side effects.

Silver-releasing antibacterial hydrogels of water-soluble polyethylene glycol (PEG) polymers with contain reactive catechol moieties, have been developed to inhibit bacterial growth while maintaining mammalian cell viability [8]. These hydrogels are suitable for antibacterial biomaterial coatings and tissue adhesives, providing a solution for implant-associated infections and improving surgical outcomes.

Mussel-inspired polydopamine hydrogel with in-situ silver nanoparticles has exhibited antibacterial activity, making it promising for antibacterial bone defect repair [53]. This hydrogel promotes bone generation and inhibits bacteria such as Staphylococcus aureus and Escherichia coli, offering the potential for addressing implant-related infections.

## 6. Optical Photothermal and Conductive Properties

Advancements in materials science and biomedical engineering have led to the development of bioinspired polymers with significant optical, photothermal, conductive, and sensing properties. These innovative materials hold great potential for various applications in the biomedical field.

One area of research involves the encapsulation of semiconducting polymer nanoparticles (SPNs) using biocompatible polydopamine (PDA). PDA-encapsulated SPNs have demonstrated enhanced structural stability, brightness for photoacoustic imaging, and photothermal therapy efficacy [54]. This technology offers potential as a theranostic agent with applications in imaging and therapy.

Another promising strategy is the use of functionally graded multilayers (FGMs) for bioinspired dental crown structures. By mimicking the dentin-enamel junction, FGMs improve the deformation behavior of dental crowns. Understanding the effects of layer thickness and architecture provides valuable insights into the design and optimization of these structures [55].

Surface modification with polydopamine (PDA) has shown tremendous potential in the field of theranostics. PDA serves as a versatile surface modifier for chemo-photothermal therapy and tumor imaging. The advantages and strategies of PDA-based nanosystems in theranostic applications have been extensively studied [56].

Bioinspired polymers have also been investigated for their optical and photothermal properties. For example, the functionalization of light-responsive polymer gels with photo-responsive chromophores enables shape reconfiguration and motion, including self-sustained motion. This research provides guidelines for controlling gel-based materials’ shape and movement, making them promising candidates for various applications [24].

The development of biomimetic surface modification of silica nanoparticles (SiO_2_ NPs) with synthetic polymers have paved the way for multifunctional biomedical applications. One-step strategies using hydrophilic polymers have been employed to achieve high water dispersibility, low cytotoxicity, and controlled drug release, making them highly desirable for biomedical applications [57].

In addition to their optical and photothermal properties, bioinspired polymers have shown excellent conductive characteristics. Mussel-inspired polydopamine (PDA) modification has been utilized to create functionalized and electroconductive polymeric matrices for bone tissue engineering. These modified matrices have improved mechanical properties, hydrophilicity, bioactivity, and cell-scaffold interactions [58].

Finally, the progress and potential of heparin-based and heparin-inspired hydrogels in biomedical applications cannot be overlooked. These hydrogels have been investigated for their optical and sensing capabilities, making them promising for biosensors and controlled drug release applications [25].

## 7. Cancer

The field of cancer research has witnessed significant advancements in recent years, particularly in the development and application of bioinspired polymers for anticancer strategies. Bioinspired polymers offer unique properties in cancer applications and functionalities that make them highly suitable for various aspects of cancer treatment, including drug delivery, imaging, and therapy.

One area of significant interest involves the surface modification of nanocarriers with polydopamine (PDA) for chemo-photothermal therapy and imaging. The advantages of PDA as a coating material for different nanomaterials enable the development of theranostic capabilities, improved drug loading, and controlled release behavior, making it an efficient platform for cancer treatment [56].

**Figure 5:**
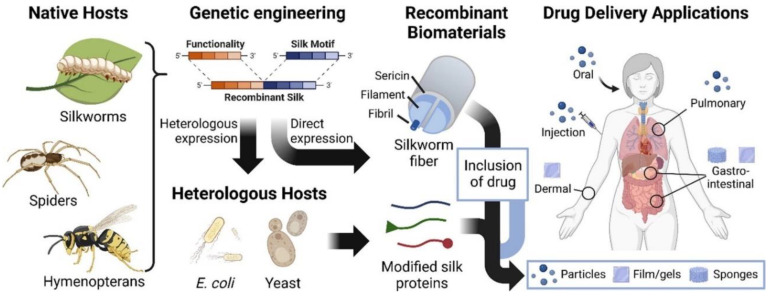
Native sources for silks, the design, and the formulation of recombinant silk biomaterials as drug delivery systems [11].

The design and formulation of recombinant silk biomaterials as drug delivery systems (DDSs) with bioinspired silk materials possess desirable properties such as processability, self-assembly, drug loading capacity, and degradability, making them excellent candidates for personalized drug delivery systems in cancer therapy [11]. Figure 5 shows an overview of native sources for silks and silk sequences, and the design as well as formulation of recombinant silk biomaterials as drug delivery systems in different formats, such as hydrogels, porous sponges, films, or particles.

Furthermore, bioinspired polymers have been explored for surface modification of silica nanoparticles (SiO_2_ NPs) to enhance their performance in biomedical applications. The one-step strategy with high water dispersibility, low cytotoxicity, and controllable drug loading and release behavior has demonstrated great potential for multifunctional biomedical applications, including cancer therapy [57].

In addition to surface modification, the development of bioinspired polymer-based nanocarriers have shown promise in tumor-specific drug delivery and effective release. The combination of lipid carriers and functional polymers in lipid-based drug delivery systems (LBDDSs) has the potential to advance cancer therapies through enhanced drug encapsulation and controlled release mechanisms [59].

Moreover, biodegradable rod-like polymer micellar systems have been designed as efficient drug delivery vehicles for anticancer agents. These bioinspired systems exhibit long blood circulation, high cellular internalization, and improved therapeutic efficacy against cancers, addressing the challenges associated with drug delivery to tumor sites [60].

The conjugation of bioinspired polymers with specific targeting ligands has also shown significant potential in cancer therapy. For instance, the conjugation of folate to noscapine, a tubulin-binding anti-cancer agent, enhances its activity against ovarian epithelial cancers overexpressing folate receptor alpha, thereby improving its efficacy as a potential treatment option [27].

## 8. Tissue Engineering

In recent years, the field of tissue engineering has witnessed meaningful advancements through the utilization of bioinspired polymers. These polymers, inspired by natural substances and processes, offer unique properties and functionalities that make them highly suitable for numerous applications in regenerative medicine. The application of bioinspired polymers in tissue engineering has shown promise, specifically in cartilage and bone tissue regeneration, spheroid fabrication, wound healing, and heart valve tissue engineering.

One notable application of bioinspired polymers is the development of a hyaluronic acid (HA)-based bioink for 3D bioprinting of cartilage tissue constructs. This bioink demonstrates suitable printability, biocompatibility, and biodegradability, making it an ideal candidate for cartilage tissue engineering [61].

Another advancement lies in the functionalization of a polymeric matrix with mussel-inspired polydopamine (PDA) for bone tissue engineering. The incorporation of PDA enhances cell adhesion, proliferation, and differentiation of bone marrow mesenchymal stem cells, showcasing promising cell-scaffold interactions and its potential for reconstructive bone substitutes [58].

Elastin-like polypeptides (ELPs) have emerged as a versatile substrate for spheroid fabrication in 3D cell culture. These bioinspired polymers provide a cost-effective, high-throughput, and reproducible alternative for spheroid culture, catering to a wide range of applications in tissue engineering [28].

The integration of bioinspired silica-collagen materials has demonstrated diverse structures, bioactivity, cytocompatibility, and controlled drug delivery potential. These materials hold great promise for biomedical applications and the development of innovative biomedical devices in tissue engineering [62].

Surface modification of biomaterials with polydopamine (PDA) has been proven to be a versatile technique inspired by mussel adhesive proteins. This modification enhanced substrate properties and enabled biomolecule immobilization, offering potential for tissue regeneration in various tissue engineering applications [63].

Collagen-based biocomposites have shown significant potential for bone and cartilage regeneration. These bioinspired polymers improve mechanical properties and osteogenic activities. By combining collagen with natural/synthetic polymers, bioactive ceramics, or carbon-based materials, collagen-based biocomposites can be tailored as scaffolds, delivery platforms, and proangiogenesis agents, advancing tissue engineering approaches [64].

Furthermore, bioinspired polymers have been employed in the development of a mussel-inspired bioactive scaffold for diabetic wound healing. The modification of a collagen and hyaluronic acid scaffold with polydopamine exhibits antioxidant effects, regulation of inflammation, and drug loading/release capabilities, providing potential for the treatment of diabetic wounds [65].

In the field of heart valve tissue engineering, anisotropic PEG-based hydrogels have shown promise, with the use of bioinspired polymers that mimic the mechanical behavior and biological functions of native heart valve leaflets. By employing photolithographic patterning, anisotropic mechanical properties can be established, offering potential solutions for heart valve tissue engineering [19].

## 9. Wound Healing

In the realm of wound healing and wound dressing applications, bioinspired polymers have emerged as promising solutions, to include oriented antibacterial sericin microneedles, supramolecular polymer hydrogels, MXene-based microneedle dressings (MMN), helical microfiber composite membranes, and mussel-inspired bioactive scaffolds.

One notable advancement is the development of oriented antibacterial sericin microneedles for enhanced wound healing. Derived from silkworm cocoons, these microneedles promote skin repair by stimulating hair follicle regeneration and angiogenesis. The incorporation of zinc oxide nanoparticles enhances their antibacterial properties, making them particularly effective in healing infected wounds [66].

Inspired by the remarkable mechanical properties of sea cucumbers, supramolecular polymer hydrogels have been developed. These hydrogels exhibit stimuli-responsive mechanical properties and can modulate their network stability and properties through pH and calcium ion concentration. Such bioinspired hydrogels hold great promise for wound healing applications [51].

Wound dressing has witnessed the emergence of MXene-based microneedle dressings (MMN). Inspired by the wrinkles and villi structure of the intestine, these ductile and highly conductive dressings enable controllable drug delivery and continuous motion sensing through near-infrared (NIR) irradiation. The integration of MXene-based microneedle dressings shows potential for intelligent wound management [67].

For joint wound dressings, a novel solution comes in the form of helical microfiber composite membranes. These membranes combine alginate-based helical fibers with a polyacrylamide/gelatin (PAM-Gel) matrix, resulting in composite polymer membranes with excellent mechanical properties. With the ability to offer controlled drug release and monitor joint motion, these membranes present a cost-effective solution for functional joint wound dressings [39].

**Figure 6:**
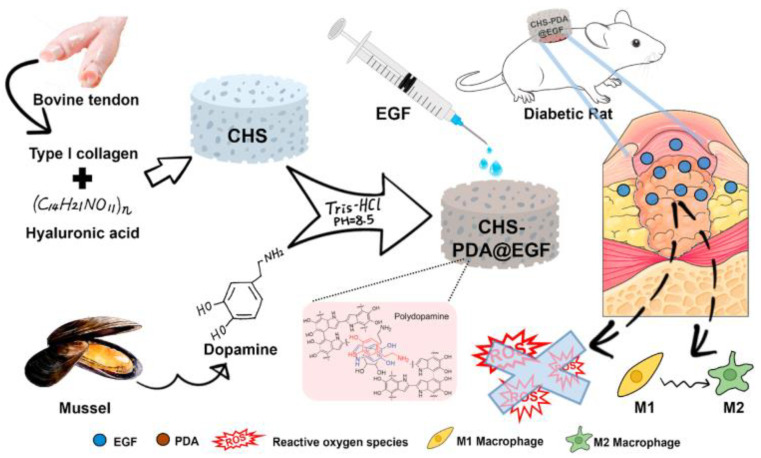
Schematic illustration of the production processes of the CHS-PDA @EGF and the mechanism of CHS-PDA@EGF for promoting chronic wound healing on the full-thickness wound in the diabetic Male Sprague–Dawley Rat model [65].

In addressing the specific needs of diabetic wounds, a mussel-inspired bioactive scaffold has shown promise. This scaffold is composed of collagen and hyaluronic acid, modified with polydopamine, and loaded with endothelial growth factor (EGF). It exhibits properties that promote wound healing, reduce inflammation, and address the unique challenges associated with diabetic wounds. Such mussel-inspired bioactive scaffolds hold potential as low-cost wound dressings for diabetic patients [65]. Figure 6 shows EGF-loaded collagen-hyaluronic acid (CHS)- polydopamine nanoparticles (PDA) may be a proper choice for clinical tissue engineering applications to improve chronic wound repair.

Table 1 summarizes various bioinspired polymers that have been prepared and studied for diverse applications in biomedicine and regenerative medicine.

## 10. Structure-Property Correlation

Biomimetic and bioinspired polymers represent a captivating frontier at the intersection of materials science and nature’s ingenuity. These cutting-edge materials, synthesized and polymerized using nature’s blueprints, offer a myriad of possibilities with their unique properties [68,69,70,71,72,73,74,75,76].

### 10.1. Synthesis

- Synthesis, Reaction, Polymerization, and Crosslinking: The advancements in bioinspired polymers have been facilitated by breakthroughs in synthesis, reaction, polymerization, and crosslinking methods [13]. These techniques allow researchers to precisely control the molecular architecture and chemical composition of the polymers, which directly impacts their final properties. Tailoring the polymer structure at the molecular level enables the development of materials with unique functionalities for specific biomedical applications.
- Dual Functional Coatings, Biohybrid Nanoreactors, Shell- and Core-Crosslinked Nanoparticles: The use of dual functional coatings, biohybrid nanoreactors, and shell- and core-crosslinked nanoparticles has shown promise in preventing biofilm formation, combating infections, enhancing drug release, and improving stability [6,12,13]. These design strategies impart specific chemical and physical characteristics to the bioinspired polymers, making them suitable for targeted biomedical applications.
- Mussel-Inspired Chemistry and Catechol Chemistry: Mussel-inspired chemistry and catechol chemistry have been utilized in bioinspired polymer design. These approaches mimic the adhesive properties found in mussel proteins, leading to enhanced surface adhesion, biocompatibility, and biodegradability of the polymers. This enables their use in tissue engineering, wound healing, and drug delivery applications [14,15,16].
- Tannic Acid-Based Surface Modification: Tannic acid-based surface modification has emerged as an effective method for modifying bioinspired polymers. It imparts antifouling properties to the materials, making them ideal for marine and biomedical applications, where inhibiting fouling is crucial for device performance [17,31].
- Applications in Biomedical Materials and Healthcare: The mentioned bioinspired polymers find applications in various biomedical fields, such as implantable biomedical devices, tissue engineering, wound healing, drug delivery, and biosensors. Their specific structures contribute to improved biocompatibility, controlled drug release, and targeted therapies, thus advancing targeted and personalized medicine [13,14,16,29,31,32,39,41,51].
- Hydrophilic Magnetic Nanorods, Amphiphilic Copolymer Micelles, Biomimetic Superlubricated Nanoparticles, MoS2-Based Polymer Nanocomposites: Further advancements in bioinspired polymers have led to the development of hydrophilic magnetic nanorods, amphiphilic copolymer micelles, biomimetic superlubricated nanoparticles, and MoS2-based polymer nanocomposites [77,78,79,80,81]. These novel structures offer potential applications in drug pollution removal, drug carriers, osteoarthritis treatment, and photothermal cancer treatment, expanding the possibilities for various biomedical interventions.

### 10.2. Adhesion

- Catechol-Functionalized Patches for Transdermal Drug Delivery: Catechol-functionalized patches demonstrate excellent adhesion, making them promising candidates for transdermal drug delivery systems [5]. The incorporation of catechol groups enables strong adhesion to the skin, enhancing the efficiency of drug delivery through this route. The specific structure of the catechol-functionalized polymers plays a crucial role in their adhesive properties.
- Nature-Inspired Dual Functional Coatings for Implantable Biomedical Devices: Nature-inspired dual functional coatings show potential in improving the performance of implantable biomedical devices [6]. These coatings can prevent biofilm formation and infections, which are directly related to their chemical composition and surface structure. The design of these polymers is crucial in achieving effective adhesion and long-term performance in biomedical applications.
- Mussel-Inspired Catechol-Modified Biomaterials in Tissue Engineering: Mussel-inspired catechol-modified biomaterials find applications in tissue engineering and regenerative medicine [7]. Their adhesive properties facilitate tissue regeneration and drug delivery to the targeted site. The molecular structure of the catechol-modified biomaterials influences their adhesion to biological tissues, making them suitable for specific biomedical applications.
- Silver-Releasing Hydrogels with Mussel-Inspired Catechol Moieties: Silver-releasing hydrogels containing mussel-inspired catechol moieties possess antibacterial properties, contributing to their suitability for various biomedical applications [8]. The presence of catechol moieties affects the hydrogel’s adhesive properties and enhances its antimicrobial effectiveness.
- Mussel-Inspired Ionoelastomers with Exceptional Adhesion: Mussel-inspired ionoelastomers exhibit exceptional adhesion, self-healing, and sensing capabilities [33]. Their unique structure and chemical composition enables strong adhesion to various surfaces, making them versatile in coatings, adhesives, drug delivery, and biomedical engineering applications.
- Recombinant Silk-Based Drug Delivery Systems: Recombinant silk-based drug delivery systems are designed to improve the loading, release, and stability of drugs, ensuring efficient and safe medical treatments [11]. The silk-based polymer’s structure and functional groups are crucial in controlling drug release and enhancing adhesive interactions at the target site.
- Tannin-Inspired Gelatin Bioadhesives for Wound Closure: Tannin-inspired gelatin bioadhesives offer wet tissue adhesion, degradation control, and cost-effectiveness, making them suitable for wound closure and tissue sealant applications [9]. The specific structure of the gelatin-based polymer plays a key role in its adhesion properties and biocompatibility.
- Progress in Other Bioinspired Polymers and Technologies: The advancements in helical microfiber composite membranes, gecko-inspired medical adhesives, polydopamine chemistry for oral medicine, chemically defined surface coatings for stem cell culture, tannic acid-based surfaces for marine applications, and mPEG-DOPA3 for biofouling issues contribute to progress in regenerative medicine, marine coatings, and biomedical devices [17,39,40,41,42,43]. The structural characteristics of these bioinspired polymers are crucial in determining their adhesion, biocompatibility, and performance in various applications.

### 10.3. Mechanical Properties

- Fmoc-RGD/Chitosan Composite Hydrogel for Tissue Engineering and Regenerative Medicine: Fmoc-RGD/chitosan composite hydrogel exhibits potential in tissue engineering, regenerative medicine, antimicrobial applications, and nanotechnology, with its mechanical properties, antibacterial activity, and silver nanoparticle production expanding its impact [18]. The specific composition and structure of this composite hydrogel influences its mechanical strength, biocompatibility, and antimicrobial properties, making it valuable for various biomedical applications.
- Polymer-Colloid Composites for Advanced Materials with Specific Structures and Properties: Polymer-colloid composites provide control over aggregate formation, benefiting advanced materials with specific structures and properties, thereby advancing non-equilibrium self-assembly and complex systems understanding [82]. The specific composition and arrangement of polymer-colloid composites allow for precise control over their mechanical properties and self-assembly behavior, making them versatile in designing complex materials.
- Functionally Graded Multilayers for Enhanced Dental Restorations: Functionally graded multilayers inspired by the dentin-enamel junction enhance the mechanical performance and longevity of dental restorations, optimizing multilayered systems for improved clinical outcomes [55]. The design of these multilayers allows for gradual variations in mechanical properties, replicating the natural tooth structure and enhancing the durability of dental restorations.
- Stimuli-Responsive SP Hydrogels for Intestinal Drug Delivery and Suturing Small Vessels: Stimuli-responsive SP hydrogels show promise in intestinal drug delivery and suturing small vessels, with their pH and calcium responsiveness, thermoplasticity, and self-healing ability making them valuable for biomedical applications [51]. The responsiveness of these hydrogels to pH and calcium ions influences their drug release behavior and their suitability for specific medical applications.
- Liquid-Free Ionoelastomers Inspired by Mussels for Versatile Applications: Liquid-free ionoelastomers inspired by mussels find applications in coatings, adhesives, and biomedical engineering, offering opportunities in various fields [33]. The specific structure and composition of these ionoelastomers enhance their adhesion properties and make them suitable for versatile applications, including biomedical devices.
- Silk-Based Drug Delivery Systems for Controlled Release and Biocompatibility: Silk-based drug delivery systems protect and control drug release, advancing effective drug delivery systems and biomaterials in healthcare [11]. The structure of silk-based polymers allows for controlled drug release, protecting the encapsulated drug and ensuring biocompatibility with the body.
- Bio-Inspired Silica-Collagen Materials for Enhanced Bioactivity and Drug Delivery: Bio-inspired silica-collagen materials enhance bioactivity, biocompatibility, and drug delivery capabilities in biomedical devices, contributing to the field of biomedicine [62]. The specific incorporation of silica and collagen influences the bioactivity and drug-loading capacity of these materials, making them valuable for biomedical applications.
- Kneading-Dough-Inspired Method for Tunable Properties and Self-Healing Hydrogel Networks: The kneading-dough-inspired method for hydrogel networks benefits drug delivery systems and biosensors, with the PEI/PAM composite hydrogel offering tunable properties and self-healing ability [20]. The specific method of preparation allows for precise control over the mechanical properties of hydrogel networks, making them valuable in drug delivery and biosensing applications.
- Citrate-Based Tissue Adhesives (POEC-d) for Strong Wet Adhesion and Biodegradability: Citrate-based tissue adhesives (POEC-d) with strong wet adhesion, biodegradability, and desirable mechanical properties advance surgical procedures and have potential as tissue adhesives and sealants [36]. The specific citrate-based composition enhances the adhesive properties and biocompatibility of these tissue adhesives, making them suitable for various biomedical applications.
- Stable Alginate Hydrogel Fibers for Tissue Engineering and Biomedical Applications: Stable alginate hydrogel fibers enable tissue engineering and biomedical applications, advancing tissue engineering techniques [15]. The specific structure and mechanical stability of these alginate hydrogel fibers make them suitable for scaffold fabrication and tissue regeneration.
- Anisotropic PEG-Based Hydrogels for Heart Valve Tissue Engineering: Anisotropic PEG-based hydrogels hold promise for heart valve tissue engineering, contributing to scaffold design for improved regenerative therapies [19]. The anisotropic design of these hydrogels allows for tissue-specific organization and function, making them valuable in heart valve tissue engineering.

### 10.4. Antifouling and Antibacterial Properties

- Poly(oxonorbornene)-based SMAMPs for Antibacterial Applications: Poly(oxonorbornene)-based SMAMPs (Synthetic Mimics of Antimicrobial Peptides) demonstrate potential as antimicrobial agents, addressing antibiotic resistance [52]. The specific structure of these SMAMPs plays a crucial role in their antibacterial activity, allowing them to effectively target and disrupt bacterial membranes.
- Bioinspired Dual Functional Coatings for Biomedical Devices: Bioinspired dual functional coatings improve the biocompatibility and long-term performance of implantable biomedical devices by reducing bacterial adhesion and protein adsorption [53]. The design of these coatings, inspired by natural systems, influences their surface properties, making them resistant to bacterial attachment and biofilm formation.
- Glucose-Responsive Nanoreactors with Antimicrobial Enzymes: Glucose-responsive nanoreactors loaded with antimicrobial enzymes show promise for therapeutic applications against drug-resistant bacterial infections [12]. The structure of these nanoreactors enables them to respond to specific environmental cues (e.g., glucose concentration) and release antimicrobial enzymes at the target site.
- Fmoc-RGD/Chitosan Composite Hydrogel for Tissue Engineering: Fmoc-RGD/chitosan composite hydrogel serves as a versatile scaffold for tissue engineering, axonal regeneration, and drug delivery, offering a multifunctional platform for biomedical applications [18]. The composition and structural characteristics of this composite hydrogel influence its mechanical properties, biocompatibility, and drug-loading capacity.
- Phenylalanine-Based Metal-Biomolecule Complexes and Cu(mDF) Coordination Polymer for Water Purification: Phenylalanine-based metal-biomolecule complexes and Cu(mDF) coordination polymer address water purification challenges by detecting and removing organic pollutants [21]. The coordination and complexation of metals with the phenylalanine-based biomolecules play a key role in the removal of pollutants, making them effective water purification agents.
- Titanium-Coated Dendritic Material for Implant-Associated Infection Treatment: Titanium-coated dendritic material enhances implant-associated infection treatment by concentrating and activating antibiotic prodrugs locally [83]. The specific structure of the dendritic material and the titanium coating contributes to its ability to release antibiotics in a controlled manner, improving antibiotic therapy effectiveness while minimizing toxicity.
- Virus-Inspired Nanodrugs (VNDs) for Enhanced Antibiotic Delivery: Virus-inspired nanodrugs (VNDs) provide a biomimetic approach to enhance antibiotic delivery and efficacy against bacteria [84]. The structure of VNDs mimics virus-like particles, allowing them to efficiently deliver antibiotics to bacterial cells and address challenges related to bioavailability and antibiotic resistance.

### 10.5. Optical Photothermal and Conductive Properties

- Encapsulation of Semiconducting Polymer Nanoparticles (SPNs) with Polydopamine (PDA) for theranostic. The encapsulation of semiconducting polymer nanoparticles with polydopamine offers a versatile platform for the development of functionalized nanoparticles with enhanced stability and bioconjugation capabilities, advancing theranostic [54]. The specific structure of the SPNs and the polydopamine shell influences their stability and surface chemistry, allowing for efficient bioconjugation and targeted therapeutic and diagnostic applications.
- Bio-Inspired Polymers in Drug Delivery Systems for Diabetes Management: Bio-inspired polymers in drug delivery systems contribute to advancements in the targeted management of diabetes and its complications [85]. The design of these polymers plays a crucial role in their drug-loading and release capacities, enabling specific and controlled drug delivery against diabetes-related conditions.
- Functionally Graded Multilayers for Enhanced Dental Restorations: Functionally graded multilayers that mimic the dentin-enamel junction provide enhanced mechanical performance for dental materials and restorative dentistry [55]. The specific composition and structure of these multilayers allow them to replicate the natural tooth structure, resulting in improved mechanical properties and longevity of dental restorations.
- Polydopamine Surface Modification for Nanomedicine Applications: Polydopamine surface modification of nanosystems enables targeted drug delivery, photothermal therapy, and tumor imaging, advancing nanomedicine and personalized medicine [56]. The polydopamine layer plays a critical role in facilitating targeted drug delivery and enhancing the therapeutic and imaging capabilities of nanosystems.
- Surface-Modified SiO2-poly(PEGMA-IA-DA) Nanoparticles for Multifunctional Biomedical Applications: Surface-modified SiO2-poly(PEGMA-IA-DA) nanoparticles exhibit desirable properties for targeted drug delivery, imaging, and therapeutics, advancing multifunctional biomedical applications [57]. The specific surface modifications and polymer structure enable these nanoparticles to carry out multiple functions, making them versatile tools in various biomedical applications.
- Poly(dopamine) (PDA) Coatings for Cell Interfacing, Drug Delivery, and Biosensing: Poly(dopamine) (PDA) coatings find diverse applications in cell interfacing, drug delivery, and biosensing, transforming bioengineering and healthcare systems [86]. The structure of the PDA coatings plays a key role in their biocompatibility, drug-loading capacity, and sensing capabilities.
- Electrochemical Biosensor for Neurobiology and Drug Discovery: The developed electrochemical biosensor offers a low-cost, sensitive, and selective approach for detecting acetylcholinesterase (AChE) activity and screening potential inhibitors, impacting drug discovery and neurobiology [26]. The design of the biosensor’s sensing elements and the electrode surface influences its sensitivity and specificity in detecting AChE activity, making it valuable in neurobiological research and drug development.

### 10.6. Cancer

- Eco-Friendly Chitosan/Copper Oxide Nanocomposites for Anticancer Therapy: Eco-friendly synthesis of chitosan/copper oxide nanocomposites using rutin shows potential as an anticancer agent, contributing to the advancement of nanomedicine [87]. The specific structure and composition of these nanocomposites influence their biocompatibility, drug-loading capacity, and anticancer activity.
- Nanoscale Coordination Polymers for Breast Cancer Drug Delivery: Nanoscale coordination polymers offer promising applications in anticancer drug delivery, improving chemotherapy outcomes specifically in breast cancer treatment [88]. The coordination and composition of these polymers play a crucial role in their drug-loading capacity and targeted delivery to breast cancer cells.
- pH-Responsive Polydopamine (PDA) Capsules for Controlled Drug Release: pH-responsive polydopamine (PDA) capsules provide a controlled drug release mechanism, enhancing anticancer drug delivery and stimuli-responsive systems [89]. The pH-sensitive nature of PDA capsules allows them to release drugs in response to specific acidic conditions in the tumor microenvironment, improving therapeutic efficacy.
- Nanocarriers Modified with Polydopamine and Sensitive to Near-Infrared Light for Chemo-Photothermal Therapy and Imaging: Nanocarriers modified with polydopamine and sensitive to near-infrared light have applications in chemo-photothermal therapy and tumor imaging, advancing nanosystems with diagnostic and therapeutic capabilities [56]. The combination of polydopamine modification and light sensitivity enables targeted drug delivery and localized photothermal therapy for cancer treatment.
- Silk-Based Drug Delivery Systems for Controlled and Targeted Delivery: Silk-based drug delivery systems offer controlled and targeted drug delivery with reduced side effects, impacting biomedical applications [11]. The silk-based polymer’s structure allows for controlled drug release, improving the efficiency of drug delivery and reducing unwanted side effects.
- Functional Polymers Integrated with Lipid-Based Drug Delivery Systems for Tumor-Specific Delivery: Functional polymers integrated with lipid-based drug delivery systems enable tumor-specific delivery and controlled drug release, improving cancer therapy [59]. The design of these polymers and lipid carriers facilitates specific targeting to tumor cells and controlled release of therapeutic agents.
- Rod-Like Nanocarriers for Enhanced Drug Delivery Efficiency: Rod-like nanocarriers enhance drug delivery efficiency and therapeutic efficacy against cancer, impacting future clinical applications [60]. The unique rod-like structure of these nanocarriers allows for increased drug-loading capacity and efficient cellular uptake, leading to improved therapeutic outcomes.
- Innovative Strategies for Targeted Drug Delivery: Advancements in cancer therapy include innovative strategies such as Targetin conjugated with a folate group for enhanced targeted delivery and guanidylated chitosan-copper chelates for drug-resistant lung cancer treatment [27,90]. The specific modifications and conjugations in these strategies enable precise targeting of cancer cells and overcoming drug resistance.
- CuO-NiO@PDA-PTX/FA Nanocarriers for Breast Cancer Therapy: The development of CuO-NiO@PDA-PTX/FA nanocarriers offers tumor-targeting capability and sustained drug release for breast cancer therapy, improving treatment outcomes [91]. The combination of metal nanoparticles, PDA coating, and folate targeting enhances drug delivery specificity and sustained release, leading to improved therapeutic effects.
- Light-Controlled “Trojan Horse” Strategy for Efficient Drug Delivery: The light-controlled “Trojan horse” strategy emerges as a powerful approach for efficient and targeted drug delivery, advancing drug delivery systems in cancer therapy [92]. The design of this strategy allows for controlled drug release upon light activation, enabling precise targeting and delivery to cancer cells.
- Dopamine-Based Molecular Imprinting Polymers for Drug Analysis and Diagnostics: Dopamine-based molecular imprinting polymers provide a selective electrochemical sensor for drug analysis and clinical diagnostics [30]. The specific molecular imprinting in these polymers allows for highly selective detection of drugs, making them valuable tools for drug analysis and diagnostics.

### 10.7. Tissue Engineering

- HA-Based Bioink for Cartilage Tissue Engineering in 3D Bioprinting: The use of HA-based bioink in 3D bioprinting shows promise for cartilage tissue engineering, providing an optimal niche for chondrocyte growth and potential treatment for osteochondral disorders [61]. The structure of the HA-based bioink influences its biocompatibility, mechanical properties, and ability to support chondrocyte growth and cartilage regeneration.
- Functionalized and Electroconductive Scaffolds for Bone Tissue Engineering: Functionalized and electroconductive scaffolds offer improved cell-scaffold interactions, making them promising for bone tissue engineering and bone regeneration [58]. The specific functional groups and conductive properties integrated into the scaffolds enhance cellular attachment, proliferation, and osteogenic differentiation.
- Elastin-Like Polypeptides (ELPs) for Spheroid Fabrication: Elastin-like polypeptides (ELPs) provide a versatile and cost-effective platform for spheroid fabrication, advancing drug discovery, stem cell research, and tumor biology [28]. The structure of ELPs allows for controlled self-assembly into spheroids, making them valuable tools in various biomedical applications.
- Bio-Inspired Silica-Collagen Materials for Controlled Drug Delivery: Bio-inspired silica-collagen materials with controlled drug delivery capabilities are suitable for innovative biomedical devices [62]. The structure and composition of these materials allow for the controlled release of therapeutic agents, making them useful in various tissue engineering applications.
- Engineering Scaffolds with Cues from the Embryonic Tendon Microenvironment for Tendon Regeneration: Engineering scaffolds with cues from the embryonic tendon microenvironment enhances tendon regeneration, contributing to advancements in regenerative medicine [93]. The specific cues integrated into the scaffolds influence cellular responses and promote tendon tissue regeneration.
- Polydopamine-Based Surface Modification Techniques for Tissue Engineering Applications: Polydopamine-based surface modification techniques enhance tissue responses, promoting repair and immune processes in tissue engineering applications [63]. The structure of the polydopamine coating plays a key role in promoting tissue integration and regeneration.
- Collagen-Based Biocomposites for Bone and Cartilage Tissue Regeneration: Collagen-based biocomposites improve mechanical properties and osteoinductivity, promoting bone/cartilage tissue regeneration and therapeutic applications [64]. The composition and structure of these biocomposites enhance their mechanical strength and ability to support tissue regeneration.
- Mussel-Inspired Bioactive Scaffolds for Diabetic Wound Healing: The use of mussel-inspired bioactive scaffolds accelerates diabetic wound healing through antioxidant properties and inflammation regulation, addressing the challenges of non-healing diabetic wounds [65]. The mussel-inspired bioactive components contribute to improved wound healing and tissue repair.
- “Switchable Surfaces” of Microfibrous Scaffolds for Targeted Cell Recruitment: “Switchable surfaces” of microfibrous scaffolds enable targeted cell recruitment, advancing regenerative medicine and tissue engineering [48]. The design of these switchable surfaces allows for controlled release of bioactive factors, attracting specific cell types for tissue repair and regeneration.
- Anisotropic PEG Hydrogels for Heart Valve Tissue Engineering: Anisotropic PEG hydrogels with biomimetic design features offer transformative approaches for heart valve tissue engineering, modulating cell behavior and tissue development [19]. The anisotropic design of these hydrogels mimics native heart valve structure, promoting tissue-specific organization and function.

### 10.8. Wound Healing

- Oriented Antibacterial Sericin Microneedles for Wound Healing: Oriented antibacterial sericin microneedles demonstrate their potential in promoting wound healing in infected wounds through efficient penetration, directional traction, antibacterial activity, and skin repair [66]. The specific structure and composition of these microneedles play a crucial role in their antibacterial properties, mechanical strength, and ability to promote wound closure.
- pH and Calcium Ion Responsive SP Hydrogels for Intestinal Drug Delivery: pH and calcium ion responsive SP hydrogels hold promise as biodevices for intestinal drug delivery and injectable fillers for suturing small vessels [51]. The responsiveness of these hydrogels to changes in pH and calcium ion concentration influences their drug release behavior and their suitability for specific medical applications.
- Silk-Based Drug Delivery Systems for Targeted and Controlled Release: Silk-based drug delivery systems offer targeted and controlled drug release with customizable design and reduced side effects [11]. The structure of silk-based polymers allows for controlled drug release, making them valuable tools for delivering therapeutics with precision and minimizing side effects.
- MXene-Based Microneedle Dressing for Wound Healing and Soft Robotics: MXene-based microneedle dressing with biomimetic structure and conductive pathways advances wound healing and finds applications in artificial tendons and soft robotics [67]. The specific structure of MXene-based microneedles enables efficient drug delivery, electrical conduction, and tissue regeneration.
- Alginate-Based Helical Microfiber Composite Membranes for Joint Wound Dressings: Alginate-based helical microfiber composite membranes serve as versatile joint wound dressings, promoting healing, controlled drug release, and motion monitoring [39]. The unique helical structure of these composite membranes enhances their mechanical properties and drug-loading capacity, making them suitable for joint wound dressings.
- Mussel-Inspired Bioactive Scaffold CHS-PDA-2@EGF for Chronic Diabetic Wound Healing: Mussel-inspired bioactive scaffold CHS-PDA-2@EGF accelerates chronic diabetic wound healing by reducing inflammation and promoting tissue repair [65]. The incorporation of mussel-inspired bioactive components contributes to the scaffold’s bioactivity and ability to enhance wound healing processes.

## 11. Perspective

Biomimetic and bioinspired polymers are leveraging nature’s designs to create innovative materials with unique properties [68,69,70,71,72,73,74,75,76]. These advancements have far-reaching implications across various fields, including biomedical materials, healthcare, environmental remediation, and industrial applications.

In synthesis, the development of bioinspired polymers has led to the creation of diverse materials such as dual functional coatings, biohybrid nanoreactors, and mussel-inspired chemistry [6,12,14]. These materials have shown immense potential in biomedical devices, tissue engineering, drug delivery, and biosensors, contributing to the advancement of personalized medicine [13,16,29,31,32,58,62,65]. Moreover, new materials like hydrophilic magnetic nanorods and amphiphilic copolymer micelles have expanded possibilities in drug pollution removal and photothermal cancer treatment [77,78].

The adhesion properties of bioinspired polymers have resulted in promising applications in transdermal drug delivery, implantable biomedical devices, tissue engineering, and wound closure [5,6,7,8,33]. Innovations such as gecko-inspired medical adhesives and tannic acid-based surfaces for marine applications have also shown potential in diverse areas like regenerative medicine and marine coatings [9,17,38,40].

The mechanical properties of biomimetic materials have led to advancements in tissue engineering, dental restorations, and drug delivery [18,55,82]. Additionally, anisotropic PEG-based hydrogels have shown promise for heart valve tissue engineering [19].

Antifouling and antibacterial properties of bioinspired polymers have significant applications in wound healing, tissue engineering, drug delivery, and environmental remediation [6,8,9,17,22,66]. These materials play a crucial role in combatting infections and enhancing the performance of biomedical devices.

In terms of optical, photothermal, and conductive properties, bioinspired polymers have driven progress in theranostic, targeted drug delivery, nanomedicine, biosensing, and neurobiology [54,56,57,85,86]. These advancements offer exciting opportunities for personalized medicine and diagnostic applications.

In cancer, bioinspired polymers have shown promise in targeted drug delivery, chemo-photothermal therapy, and improving treatment outcomes [56,87,88,89]. These materials have the potential to transform cancer therapeutics, enhancing drug delivery, and reducing side effects.

In tissue engineering, bioinspired materials like HA-based bioinks and functionalized electroconductive scaffolds have significant applications in cartilage tissue engineering and bone regeneration [58,61]. They contribute to advancements in regenerative medicine and tissue repair.

Finally, in wound healing, bioinspired polymers have advanced drug delivery systems, wound dressings, and tissue repair processes [11,51,66,67]. These innovations offer new avenues for promoting wound healing and improving patient outcomes.

## 12. Conclusions

The integration of bioinspired polymers, including dual functional coatings, biohybrid nanoreactors, shell- and core-crosslinked nanoparticles, mussel-inspired chemistry, catechol chemistry, and tannic acid-based surface modification, has transformed the field of biomedical materials and healthcare. These advancements have enabled the development of innovative materials and technologies with diverse applications, ranging from implantable biomedical devices and tissue engineering to drug delivery and biosensors. By harnessing the unique properties and functionalities inspired by nature, bioinspired polymers have significantly improved targeted and personalized medicine, addressing various healthcare challenges and paving the way for enhanced healthcare outcomes. These materials offer promising solutions for preventing biofilm formation, combating infections, improving drug release, and advancing stability, making them valuable tools in biomedical sciences and related industries.

Disclosure: The authors partly used OpenAI’s large-scale language-generation model. The authors reviewed, revised, and edited the document for accuracy and take full responsibility for the content of this publication. The authors used Bing AI image creator to draw the graphical abstract.

## Figures and Tables

**Table 1:** Bioinspired Polymers for Biomedicine and Regenerative Medicine Applications.

Application	Bioinspired Polymer	Key Findings	Ref.
Adhesion	DMA-MPC copolymers with antimicrobial silver nanoparticles	Catechol-containing copolymers reduce bacterial adhesion and resist BSA adsorption. Successful surface modification with antimicrobial silver nanoparticles.	[6]
	Poly(hydroxyethyl acrylate-co-itaconic acid)-catechol (PHI-cat) adhesive	PHI-cat adhesive exhibits superior adhesion to human skin. Good stability, drug-polymer interaction, and adhesive performance.	[5]
	Catechol-containing hydrogel	The developed hydrogel covalently crosslinks with simultaneous silver reduction, enabling sustained silver release for at least two weeks. It exhibits antibacterial properties without significant effect on mammalian cell viability. The hydrogel films show resistance to bacterial and mammalian cell attachment.	[8]
	Recombinant silk biomaterials for drug delivery systems	The design and formulation of recombinant silk biomaterials for drug delivery systems (DDSs). It emphasizes the versatility of silk-based DDSs in various formats and highlights their properties and advantages, including processability, drug loading capacity, and degradability.	[11]
	Tannin-inspired gelatin bioadhesives	The tannin-inspired gelatin bioadhesives demonstrate excellent wet tissue adhesion strength and controllable degradation. They exhibit good cytocompatibility and possess inherent antibacterial and antifungal properties through the inclusion of tannic acid and silver nanoparticles.	[9]
	Functional polymer brushes on SS surface	The cationic polymer brushes exhibit bactericidal properties, while the zwitterionic coatings resist bacterial adhesion. The functional polymer-grafted surfaces show significant reduction in microalgal attachment (microfouling) and barnacle cyprid settlement (macrofouling) compared to the pristine SS surface.	[17]
	Poly(hydroxyethyl acrylate-co-itaconic acid)-catechol (PHI-cat) adhesive	PHI-cat adhesive exhibits superior adhesion to human skin. Good stability, drug-polymer interaction, and adhesive performance.	[5]
	Catechol-containing hydrogel	The developed hydrogel covalently crosslinks with simultaneous silver reduction, enabling sustained silver release for at least two weeks. It exhibits antibacterial properties without significant effect on mammalian cell viability. The hydrogel films show resistance to bacterial and mammalian cell attachment.	[8]
	Polydopamine (PDA) nanosheets	The 2D mussel-inspired PDA nanosheets and their polymer-brush derivatives show lateral integrity and robustness, promoting cell growth and attachment. A PDA-based poly(3-sulfopropyl methacrylate) carpet exhibits nonfouling behavior.	[34]
	Citrate-based tissue adhesives (POEC-d)	POEC-d adhesives with PEO to 1,8-octanediol mole ratio of 70% exhibited water solubility. They showed rubber-like behavior, a degradation rate of 1–2 weeks, and lap shear adhesion strength surpassing commercial fibrin glue. Low cytotoxicity was observed.	[36]
	Synthetic polymer-based dry adhesives	Adhesives show anisotropic adhesion properties similar to gecko adhesive system. Adhesion forces increase with lower tilt angles. High anisotropy in frictional adhesion behavior is observed.	[2]
	Triblock copolymers with catechol-anchoring groups	Robustly anchored triblock copolymers with loop conformations exhibit excellent lubricating properties, with an extremely low friction coefficient. These polymers also inhibit cell adhesion and proliferation compared to other conformations.	[37]
	TAPE (Tannic Acid-Poly(Ethylene) glycol adhesive)	TAPE demonstrates superior water-resistant adhesion compared to commercial fibrin glue. It is biodegradable under physiological conditions.	[38]
	Alginate-based helical fiber composite membranes	The composite membrane design incorporating alginate-based helical fibers and PAM-Gel achieved low-cost preparation, excellent mechanical properties, wound healing promotion, controlled drug release, and motion monitoring ability. The membranes demonstrated good biocompatibility and potential for application in joint wound dressings.	[39]
	Gecko-inspired tissue adhesive with nanoscale pillars	The adhesive demonstrates increased interfacial adhesion strength on tissue surfaces. Optimization of pillar dimensions and dextran coating enhance performance.	[40]
	Polyhydroxyalkanoates (PHA) films with polydopamine coating	Polydopamine coating enhances adhesive properties and promotes cell attachment and proliferation. In vivo biocompatibility is improved with reduced inflammation and neovascularization.	[41]
	Functionalization of polymer substrates with VN-dimer/pDA	The surface coating supports adhesion, proliferation, and colony formation of hPSCs. Enhanced focal adhesion and cell-cell interaction promote self-renewal and pluripotency of hPSCs.	[42]
	mPEG-DOPA3 modified titanium surfaces	The mPEG-DOPA3 modified titanium surfaces significantly decrease the attachment of diatom cells and algal zoospores compared to control surfaces. The modified surfaces show the highest detachment of attached cells under flow conditions, demonstrating their potential effectiveness in marine antifouling and fouling-release applications.	[43]
	PDA-coated implant surfaces	PDA coating improves hydrophilicity, enhances adhesion, proliferation, and osteogenic differentiation of bone marrow stem cells.	[44]
	Polymer microparticles with surface textures	The fabricated microparticles exhibit controllable surface roughness and strong adhesion to intestinal mucosa. Effective drug delivery is achieved.	[45]
	PVA/GA supramolecular hydrogels	The deacetylation degree of PVA affects gelation kinetics and properties. Interactions between GA and PVA chains enhance stability and elastic modulus.	[46]
	Chitosan mixed hydrogels with catechol compounds	Incorporation of HCA significantly enhances mucoadhesion. pH-dependent swelling behavior and controlled release of catechol compounds are observed.	[47]
	Thermoswitchable microfibrous scaffold (cRGD-PNIPAM)	The microfibrous scaffold exhibited accelerated endothelial cell adhesion and spreading through integrin-cRGD interaction below the LCST (25 degrees C), and rapid detachment at 37 degrees C as the cRGD became concealed. This reversible switchable property enables precise control over cell adhesion and detachment.	[48]
	Mussel-inspired underwater adhesive polymers	Recent studies show design progress in catechol-functional polymers for enhanced underwater adhesion. Properties and applications of these polymers reviewed.	[49]
	Biohybrid composite materials	Hydrogels with tunable mechanical properties, long-term enzymatic activities, low-fouling properties, and antimicrobial activity (>7-log reduction in bacterial adherence and viability) are potential biomedical substrates.	[50]
Mechanical Properties	Fmoc-RGD/chitosan composite hydrogel	The composite hydrogel shows enhanced gelation rate, improved mechanical properties, and increased durability in cell culture. It serves as an effective scaffold for 2D and 3D cell cultures and displays notable antibacterial activity and silver nanoparticle production.	[18]
	PEI/PAM composite hydrogel	The kneading-dough-inspired method disperses hydrophobic particles into water, forming stable suspensions. PEI/PAM composite hydrogel shows improved mechanical properties and stabilization mechanism.	[20]
	Anisotropic PEG hydrogels	PEG hydrogels with anisotropic mechanical properties were achieved via photolithographic patterning, exhibiting higher tensile modulus parallel to the stripes. RGDS and PQ peptide incorporation influenced VICs’ behavior and hydrogel degradation for heart valve tissue engineering.	[19]
	Mussel-inspired catechol crosslinked alginate hydrogel fibers	Catechol-tethered alginate fibers with inter-catechol crosslinking showed enhanced mechanical strength. The gluing capability of catechol stabilized fiber interfaces, maintaining shape fidelity of 3D constructs and facilitating cell encapsulation during culture.	[15]
	pH and calcium ion-responsive SP hydrogels	SP hydrogels doped with calcium ions exhibited excellent mechanical properties, transitioning between stiff and stable states and soft, highly swollen states with autolysis based on pH and calcium ion concentration. They also demonstrated thermoplasticity, self-healability, and biocompatibility for potential use in various applications.	[51]
Antibacterial and antifouling properties	Glucose oxidase-loaded nanoreactors	The biohybrid nanomaterials exhibit antimicrobial activity against Gram-negative and Gram-positive bacterial pathogens, including drug-resistant strains. Their toxicity is optimized for safe use under physiological glucose concentrations.	[12]
	Catechol-containing hydrogel	The developed hydrogel covalently crosslinks with simultaneous silver reduction, enabling sustained silver release for at least two weeks. It exhibits antibacterial properties without significant effect on mammalian cell viability. The hydrogel films show resistance to bacterial and mammalian cell attachment.	[8]
	Fmoc-RGD/chitosan composite hydrogel	The composite hydrogel shows enhanced gelation rate, improved mechanical properties, and increased durability in cell culture. It serves as an effective scaffold for 2D and 3D cell cultures and displays notable antibacterial activity and silver nanoparticle production.	[18]
	Phenylalanine-based metal-biomolecule complexes	The phenylalanine-based metal-biomolecule complexes demonstrate subtle sequence-dependent assembly behaviors and possess a mesoporous structure with high surface area and color-shifting feature for organic pollutant detection. They also exhibit antimicrobial properties.	[21]
	Polymeric conjugates of PMX B	Different polymeric conjugates of PMX B are synthesized, and PMAG demonstrates the most promising carrier properties for delivering PMX B, preserving its antimicrobial properties and enabling controlled drug release. Conjugate with deferoxamine shows the lowest MIC against Pseudomonas aeruginosa.	[22]
	SMAMPs (Synthetic Multifunctional Antimicrobial Polymers)	Certain hydrophilic SMAMPs demonstrated ‘doubly selective’ antimicrobial activity, targeting bacteria over mammalian cells and Gram-positive over Gram-negative bacteria. One SMAMP showed improved broad-band activity. Transmission electron studies revealed damage to bacterial membranes.	[52]
	AgNPs/PDA hydrogel	AgNPs/PDA hydrogel exhibits significant antibacterial activity, promotes bone generation, and efficiently repairs maxillary bone defects in vivo.	[53]
Optical photothermal and conductive properties	Polydopamine-encapsulated semiconducting polymer	Polydopamine (PDA) encapsulation enables uniform-sized semiconducting polymer nanoparticles (SPNs) with enhanced structural stability, increased photothermal brightness, and improved photothermal therapy (PTT) efficacy for tumor ablation.	[54]
	Contact-induced stresses in bio-inspired dental multilayers	Finite element modeling and experimental analysis demonstrate the influence of thickness and architecture on contact-induced stresses in bio-inspired dental multilayers. The multilayer structure significantly affects the stress distribution, with loading rate dependence accurately predicted.	[55]
	Polydopamine surface modification of nanocarriers	Polydopamine (PDA) serves as a versatile polymer for surface modification of nanocarriers, enabling chemo-photothermal therapy (PTT) and tumor imaging. PDA-coated nanosystems based on various materials provide enhanced functionalities and theranostic capabilities.	[56]
	SiO_2_-poly(PEGMA-IA-DA) nanoparticles	A biomimetic strategy successfully coated SiO_2_ NPs with hydrophilic polymers (poly(PEGMA-IA-DA)). The resulting nanoparticles exhibited high water dispersibility, low cytotoxicity, and controlled drug loading and release behavior, showing potential for biomedical applications.	[57]
	PVA/PU-PANI electroconductive scaffolds	Homogeneous decoration of the matrixes with PDA improved tensile strength and Young’s modulus. PDA modification enhanced hydrophilicity and supported hydroxyapatite-like crystal formation. Modified scaffolds showed suitable cell viability and enhanced osteogenic markers.	[58]
	Heparin-based and heparin-inspired hydrogels	The review categorizes different forms of heparin-based hydrogels and summarizes fabrication strategies, including covalent bonding and physical conjugation methods. Biomedical applications such as implantation, tissue engineering, biosensors, and drug release are discussed.	[25]
Cancer	Polydopamine surface modification of nanocarriers	Polydopamine (PDA) serves as a versatile polymer for surface modification of nanocarriers, enabling chemo-photothermal therapy (PTT) and tumor imaging. PDA-coated nanosystems based on various materials provide enhanced functionalities and theranostic capabilities.	[56]
	SiO_2_-poly(PEGMA-IA-DA) nanoparticles	A biomimetic strategy successfully coated SiO_2_ NPs with hydrophilic polymers (poly(PEGMA-IA-DA)). The resulting nanoparticles exhibited high water dispersibility, low cytotoxicity, and controlled drug loading and release behavior, showing potential for biomedical applications.	[57]
	Ligands and lipid-based drug delivery systems (LBDDSs)	The review analyzes ligands used for tumor-specific delivery, strategies for controlled drug release in tumor microenvironments, and recent designs of LBDDSs. It highlights the potential applications of functionalized LBDDSs in cancer therapy, contributing to improved treatment outcomes.	[59]
	Rod-like biodegradable polymer micellar system	Rod-shaped micelles, with dimensions of approximately 40 nm in diameter and 600 nm in length, exhibit minimal uptake by the reticuloendothelial system (RES), prolonged blood circulation half-lives, and improved delivery efficiency compared to spherical micelles. They demonstrate superior potency and efficacy against artificial solid tumors.	[60]
	Folate-conjugated noscapine derivative (Targetin)	Targetin, a folate-conjugated noscapine derivative, shows a strong binding affinity to tubulin and alters microtubule assembly dynamics. It exhibits enhanced sensitivity compared to noscapine in various cancer cell lines, particularly ovarian cancer cells that overexpress FRalpha.	[27]
Tissue engineering	PVA/PU-PANI electroconductive scaffolds	Homogeneous decoration of the matrixes with PDA improved tensile strength and Young’s modulus. PDA modification enhanced hydrophilicity and supported hydroxyapatite-like crystal formation. Modified scaffolds showed suitable cell viability and enhanced osteogenic markers.	[58]
	Bio-inspired silica-collagen	Bio-inspired silica-collagen materials exhibit diverse structures and properties. Polymer self-assembly and inorganic condensation interplay elucidated. Biocompatible with controlled drug delivery.	[62]
	Polydopamine-modified lyophilized collagen hyaluronic acid	The CHS-PDA-2@EGF wound dressing exhibited favorable physical and chemical properties, antioxidant effects, inflammation regulation, resistance to ROS, and promoted chronic wound regeneration in diabetic rats. It also displayed excellent swelling ability, coagulation effect, and reasonable degradation.	[65]
Wound healing	Oriented antibacterial sericin microneedles	The designed microneedles efficiently penetrate and provide directional traction for wound closure. Sericin promotes skin repair through hair follicle regeneration and angiogenesis. Zinc oxide integration enhances the microneedles’ antibacterial activity.	[66]
	MXene-based microneedle dressing	The MXene-based microneedle dressing offers ductility, biocompatibility, high conductivity, controllable drug delivery, and facilitates wound healing in animal models. Its biomimetic structure, controllable drug release, and conductive pathways contribute to intelligent wound management and other biomedical applications.	[67]
	Polydopamine-modified lyophilized collagen hyaluronic acid	The CHS-PDA-2@EGF wound dressing exhibited favorable physical and chemical properties, antioxidant effects, inflammation regulation, resistance to ROS, and promoted chronic wound regeneration in diabetic rats. It also displayed excellent swelling ability, coagulation effect, and reasonable degradation.	[65]
	Alginate-based helical fiber composite membranes	The composite membrane design incorporating alginate-based helical fibers and PAM-Gel achieved low-cost preparation, excellent mechanical properties, wound healing promotion, controlled drug release, and motion monitoring ability. The membranes demonstrated good biocompatibility and potential for application in joint wound dressings.	[39]
	pH and calcium ion-responsive SP hydrogels	SP hydrogels doped with calcium ions exhibited excellent mechanical properties, transitioning between stiff and stable states and soft, highly swollen states with autolysis based on pH and calcium ion concentration. They also demonstrated thermoplasticity, self-healability, and biocompatibility for potential use in various applications.	[51]

## Data Availability

Not applicable.
